# *COQ7* defect causes prenatal onset of mitochondrial CoQ_10_ deficiency with cardiomyopathy and gastrointestinal obstruction

**DOI:** 10.1038/s41431-024-01615-w

**Published:** 2024-05-03

**Authors:** Ilaria Pettenuzzo, Sara Carli, Ana Sánchez-Cuesta, Federica Isidori, Francesca Montanari, Mina Grippa, Giulia Lanzoni, Irene Ambrosetti, Veronica Di Pisa, Duccio Maria Cordelli, Maria Cristina Mondardini, Tommaso Pippucci, Luca Ragni, Giovanna Cenacchi, Roberta Costa, Mario Lima, Maria Antonietta Capristo, Concetta Valentina Tropeano, Leonardo Caporali, Valerio Carelli, Elena Brunelli, Monica Maffei, Hodman Ahmed Sheikhmaye, Anna Fetta, Gloria Brea-Calvo, Caterina Garone

**Affiliations:** 1 Department of Medical and Surgical Sciences, Alma Mater Studiorum, University of Bologna, 40138 Bologna, Italy; 2 IRCCS Istituto delle Scienze Neurologiche di Bologna, UOC Neuropsichiatria dell’età Pediatrica, Bologna, Italy; 3 Center for Applied Biomedical Research, Alma Mater Studiorum, University of Bologna, 40138 Bologna, Italy; 4 Centro Andaluz de Biología del Desarrollo, Universidad Pablo de Olavide-CSIC-JA and CIBERER, Instituto de Salud Carlos III, Seville, 41013 Spain; 5 Medical Genetics Unit, IRCCS Azienda Ospedaliero-Universitaria di Bologna, 40138 Bologna, Italy; 6 Pediatric Anesthesia and Intensive Care Unit, Department of Woman’s and Child’s Health, IRCCS Azienda Ospedaliero-Universitaria di Bologna, Bologna, Italy; 7 Pediatric Cardiology and Adult Congenital Heart Disease Program, Department of Cardio-Thoracic and Vascular Medicine, IRCCS Azienda Ospedaliero-Universitaria di Bologna, Bologna, Italy; 8 Department of Biomedical and Neuromotor Sciences, Alma Mater Studiorum University of Bologna, Bologna, Italy; 9 Pediatric Surgery Department, IRCCS Sant’Orsola-Malpighi Polyclinic, Alma Mater Studiorum-University of Bologna, 40126 Bologna, Italy; 10 IRCCS Istituto delle Scienze Neurologiche di Bologna, Programma di Neurogenetica, Bologna, Italy; 11 Obstetric Unit, IRCCS Azienda Ospedaliero-Universitaria di Bologna University of Bologna, Bologna, Italia; 12 IRCCS Istituto delle Scienze Neurologiche di Bologna, Programma di neuroradiologia con tecniche ad elevata complessità, Bologna, Italia

**Keywords:** Diseases, Genetics

## Abstract

*COQ7* pathogenetic variants cause primary CoQ_10_ deficiency and a clinical phenotype of encephalopathy, peripheral neuropathy, or multisystemic disorder. Early diagnosis is essential for promptly starting CoQ_10_ supplementation. Here, we report novel compound heterozygous variants in the *COQ7* gene responsible for a prenatal onset (20 weeks of gestation) of hypertrophic cardiomyopathy and intestinal dysmotility in a Bangladesh consanguineous family with two affected siblings. The main clinical findings were dysmorphisms, recurrent intestinal occlusions that required ileostomy, left ventricular non-compaction cardiomyopathy, ascending aorta dilation, arterial hypertension, renal dysfunction, diffuse skin desquamation, axial hypotonia, neurodevelopmental delay, and growth retardation. Exome sequencing revealed compound heterozygous rare variants in the *COQ7* gene, c.613_617delGCCGGinsCAT (p.Ala205HisfsTer48) and c.403A>G (p.Met135Val). In silico analysis and functional in vitro studies confirmed the pathogenicity of the variants responsible for abolished activities of complexes I + III and II + III in muscle homogenate, severe decrease of CoQ_10_ levels, and reduced basal and maximal respiration in patients’ fibroblasts. The first proband deceased at 14 months of age, whereas supplementation with a high dose of CoQ_10_ (30 mg/kg/day) since the first days of life modified the clinical course in the second child, showing a recovery of milestones acquirement at the last follow-up (18 months of age). Our study expands the clinical spectrum of primary CoQ_10_ deficiency due to *COQ7* gene defects and highlights the essential role of multidisciplinary and combined approaches for a timely diagnosis.

## Introduction

Coenzyme Q_10_ (CoQ_10_) is a lipid-soluble molecule composed of a benzoquinone ring and a 10-unit polyisoprenoid chain, which inserts the molecule into the phospholipid bilayer of various cell membrane types. The redox-active benzoquinone head group is responsible for shuttling electrons from the mitochondrial complex I (NADH: ubiquinone oxidoreductase) or complex II (succinate dehydrogenase) to complex III (ubiquinol cytochrome c reductase) in the inner mitochondrial membrane [1]. CoQ_10_ also receives electrons from other mitochondrial dehydrogenases [2], being essential for various mitochondrial processes, such as pyrimidine synthesis, and participates in the plasma membrane antioxidant system, protecting cells from ferroptosis [3].

CoQ_10_ biosynthesis is an endogenous, multi-step process mediated by a set of nuclear-encoded proteins, most of them working in a complex in the inner mitochondrial membrane (the Q-complex or Q sythome). Pathogenetic variants in genes encoding proteins playing a role in the CoQ_10_ biosynthesis pathway have been demonstrated to be responsible for primary CoQ_10_ deficiency and associated with four main clinical forms: 1) steroid-resistant nephrotic syndrome (SRNS), isolated or with neurological involvement (described in cases of defects in *PDSS2*, *COQ2*, *COQ6*, or *COQ8B*); 2) encephalomyopathy, hypertrophic/dilated cardiomyopathy, lactic acidosis, and tubulopathy (caused by defects in *PDSS2*, *COQ2*, *COQ7*, or *COQ9*); 3) neonatal cardio-encephalopathies (associated with *COQ2*, *COQ4*, or *PDSS1*); and 4) pure neurological syndromes, including isolated or combined Leigh syndrome, ARCA, and refractory epilepsy (associated with *COQ2*, *COQ4*, *COQ5*, *COQ7*, or *COQ9* defects) [4]. Isolated myopathy has been only described in individuals with secondary CoQ deficiencies [5].

*COQ7* gene encodes a carboxylate bridged diiron hydroxylase, which catalyzes the head group C6-hydroxylation that converts 2-decaprenyl-6-methoxy-3-methyl-1,4-benzoquinone/quinol (demethoxy-Coenzyme Q, DMQ or DMQH_2_) to 2-decaprenyl-5-hydroxy-6-methoxy-3-methyl-1,4-benzoquinone/quinol (demethyl-Coenzyme Q, DMeQ or DMeQH_2_) [6]. Autosomal recessive pathogenic variants have been described in more than thirty families with a variable neuromuscular, neurodevelopmental, or multisystemic phenotype [7–18] (Supplementary Table 1). Here, we report clinical, imaging, molecular genetics, and biochemical findings of 2 novel compound heterozygous variants in the *COQ7* gene identified in a Bangladesh consanguineous family with two affected siblings presenting a complex multiorgan disorder whose first disease signs were at the prenatal stage with cardiomyopathy and intestinal obstruction. Our study further enlarges the *COQ7*-associated disease spectrum and adds insights into the intrafamilial variability and response to treatment.

## Material and methods

### Patients

The legal guardians of the index cases provided written informed consent. Clinical and instrumental data were collected by reviewing medical records. Phenotyping was performed by a senior pediatric neuro-geneticist with expertise in mitochondrial disorders, cardiologists, pediatric surgeons, and gynecologists.

### Muscle biopsy

An open muscle biopsy was performed, and the muscle sample was longitudinally oriented and snap-frozen in liquid nitrogen-chilled isopentane. Serial cryostat-cut sections were stained for a panel of histological and enzyme histochemical reactions as previously described [19]. For ultrastructural investigations, a small fragment of fresh tissue was fixed in 2.5% glutaraldehyde in cacodylate buffer, post-fixed in 1% OsO_4_ in the same buffer, dehydrated, and embedded in araldite. Thin sections, stained with uranyl acetate and lead citrate, were examined with a Philips 400T transmission electron microscope. Respiratory chain complexes and citrate synthase activity were determined on skeletal muscle homogenates as previously reported with minor modifications [20].

### Molecular genetics study

DNA extraction from fibroblasts was performed using DNeasy Blood & Tissue Kit (Qiagen, #69504) following the manufacturer’s instructions. Analysis of mitochondrial DNA (mtDNA) (nt 1-16569) was performed on DNA extracted from muscle specimens in P1.a by the NGS method previously described [21]. Briefly, the mtDNA was amplified in two long-range PCR using a high-fidelity Taq DNA polymerase (PrimeSTAR Max DNA Polymerase, Takara). The NGS library was prepared by xGen™ DNA Library Preparation Kits (Integrated DNA Technologies Inc, Coralville, Iowa USA) and sequenced as 150-bp paired-end reads on MiSeq System (Illumina Inc, San Diego, CA). For mtDNA haplogroup affiliations and private variants analysis, Fastq files were analyzed with MToolBox v1.2, considering over 5% heteroplasmic variants. The m.8698A>G/MT-ATP6 was validated by mini sequencing (SNaPshot™ Multiplex Kit, Thermo Fisher #4323159).

Whole exome sequencing (WES) was performed according to the Twist Human Core Exome Kit v.1.3. Library was run as 150-bp paired-ends on Illumina instrument. Reads were checked with fastp (github.com/OpenGene/fastp) and aligned with BWA (bio-bwa. sourceforge.net) to the human genome (hg38), with a mean coverage of 141X and 98.9% of the target covered at least 20×. The variants called with GATK Toolkit (v.4.1.2.0) were annotated using the Ensembl Variant Effect Predictor tool (VEP) and filtered based on the type of variant, allele frequency in the Genome Aggregation Database (gnomAD; MAF < 0.01) (https://gnomad.broadinstitute.org/) and mode of inheritance using an in-house tool.

Sanger sequencing was performed to confirm the presence of the *COQ7* variants in proband P1.a and their segregation within the family members. Regions of interest were amplified using the following primers: forward, 5′- GGTCTCCATTACCGGTCATATCT -3′ and reverse, 5′-TTGAGCAGCCCCGTTCTAG-3′, PCR product: 234 bp; forward, 5′- TGCCAGCCCTGAATCTTACA -3′ and reverse, 5′- TCTCTGCAAATGTCACTTGGT -3′, PCR product: 214 bp. To separate the wild-type and mutated alleles and analyze the variation of the sequence, PCR product for exon 6 was purified through QIAquick Gel Extraction Kit (Qiagen, #28704), inserted into the pCR 2.1-TOPO TA (Invitrogen, #450641), and amplified in DH5α Competent Cells (Invitrogen, #18265017). Ten colonies were picked, and DNA was extracted using the QIAprep Spin Miniprep Kit (Qiagen, #27106) following the manufacturer’s instructions. Sanger sequencing was performed to confirm the presence of the frameshift mutation (primer forward: 5′- GCCAGCCCTGAATCTTACAA -3′).

### Cell cultures

Skin-derived fibroblasts were maintained in a growth medium containing high glucose DMEM+GlutaMAX (Thermo Fisher, #31053028) supplemented with 10% Fetal Bovine Serum (Thermo Fisher, #10270106) and 1% Penicillin-Streptomycin (Thermo Fisher, #15140122) and incubated in a humidified 37 °C/5% CO_2_ incubator.

### Real-time RT-PCR

Total RNA was extracted using RNeasy Minikit (Qiagen, #74104) following the manufacturer’s instructions and RNA integrity was verified by agarose electrophoresis. First-strand cDNA was synthesized through Omniscript Reverse Transcription Kit (Thermo Fisher, #205113) and used as a template for quantitative RT-PCR with SYBR Green Master Mix (Applied Biosystems, #4472942). The following primers were used: *COQ7*: forward primer 5’-GGCGGTCCCTCTCAGCTTAT-3’; reverse primer 5’-GCGGTTTGCTCCATATTCGC-3’; *COQ9*: forward primer 5’-ATGAGCAGAAGCAGCAGCCTCC-3’; reverse primer 5’-CCTCGCCGCCCTGGTCTGTATA -3’; HPRT: forward primer 5’-CATTATGCTGAGGATTTGGAA-3’; reverse primer 5’- CTTGAGCACACAGAGGGCTACA-3’.

### Western Blot

Cell pellets were homogenized in 100 μl of TG lysis buffer (20 mM Tris HCl, pH 7.5, 500 mM NaCl, 1 mM EDTA, 1% Triton X-100, 10% glycerol, 1.5 mM MgCl) containing 10 μl/ml of Halt^TM^ Protease Inhibitor Cocktail (Thermo Fisher, #87786) and protein concentrations were calculated through DC Protein Assay (Biorad, #5000111). Protein lysates were separated on precast polyacrylamide gel and the following primary antibodies were used: anti-COQ7 (Santa Cruz, #376484; 1:500 in 1X Tris Buffered Saline with 1% Casein); anti-COQ9 (Santa Cruz, # 365073; 1:500 in 1X Tris Buffered Saline with 1% Casein); anti-NDUFS1 (Abcam, #ab169540; 1:1000 in 5% milk in PBS-T); anti-SDHA (Abcam, #14715, 1:1000 in 5% milk in PBS-T); anti-UQCRFS1 (Abcam, # 14746; 1:1000 in 5% milk in PBS-T); anti-mtCO1 (Abcam, #14705; 1:1000 in 5% milk in PBS-T); anti-ATP5A (Abcam, #14748; 1:2000 in 5% milk in PBS-T); anti-GAPDH (Abcam, #8245; 1:1000 in 5% milk in PBS-T). Quantification of bands was performed by using Image Lab Software.

### CoQ measurements by HPLC

Cell membranes from fibroblasts (0.5 mg protein) were disrupted by adding SDS (1% final concentration) after adding 50 pmol of CoQ_6_ as an internal standard to estimate the CoQ_10_ recovery during the extraction process. Lipids were then dispersed with an alcohol cocktail (ethanol: isopropanol 95:5, sample: alcohol cocktail ratio 1:2 v/v). Finally, hexane extraction was performed in triplicates (dispersed sample: hexane ratio 3:5 v/v) followed by 5-min centrifugation (1000 × *g*) at 4 °C. Hexane fractions were mixed and dried under a vacuum. Dried samples were reconstituted in ethanol before HPLC analysis.

CoQ_10_ content was analyzed by a C18 Reverse Phase HPLC coupled to an electrochemical detector (ECD) as described by Rodríguez-Aguilera et al. [22]. Separation was performed by a gradient method (20 mM AcNH_4_ pH 4.4 in methanol: 20 mM AcNH_4_ pH 4.4 in propanol) with a solvent mixture of 85:15 v/v and 1.2 mL/min as starting conditions. The mobile phase turned to a 50:50 ratio from minute 6 to 8, while the flow rate decreased to 1.1 mL/min.

### Oxygen consumption by Seahorse Extracellular Flux Analyzer

Oxygen consumption rate (OCR) was determined using a Seahorse XF24 Extracellular Flux Analyzer (Agilent) following the manufacturer’s instructions. Briefly, the day before the assay, 5 × 10^4^ fibroblasts were seeded onto Seahorse 24-well plates. On the day of the experiment and before the measurements, cells were washed twice with Seahorse XF base medium (bicarbonate-free medium) supplemented with 1 g/L glucose, 2 mM glutamine, and 1 mM sodium pyruvate. Cells were incubated for 1 h at 37 °C without CO_2_, in 500 µL/well of base medium. OCR was measured under basal conditions, and after the sequential injection of oligomycin (4 µM final concentration), FCCP (1 µM final concentration), and rotenone plus antimycin A (1 µM and 2.5 µM final concentration respectively). To normalize respiration rates, cells were harvested and counted after the assay.

### Statistical analysis

Statistical tests were performed with GraphPad 8 (GraphPad Prism Software). Statistical significance between two samples was determined through Unpaired Student’s *t* test, while One-Way ANOVA followed by post hoc tests was used for multiple group comparisons. Statistical significance was expressed as: *p < 0.05, **p < 0.01, ***p < 0.001 and ****p < 0.0001.

## Results

### Clinical findings

**Fig. 1:**
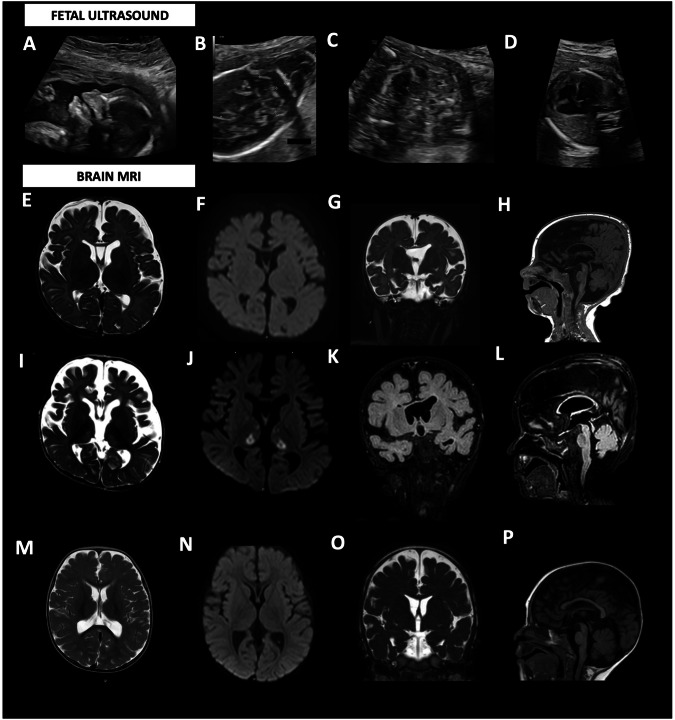
Imaging studies. **A**–**D** Ultrasound images at 20 weeks of gestation in P1.a showing dysmorphic profile (**A**), cystic hygroma (17.5 mm) (**B**), and echogenic bowel with a mildly dilated loop (**C**); and at 28 weeks gestation in P1.b showing cardiac biventricular hypertrophy, accompanied by diminished contractility in both the right and left ventricles (**D**). Patient 1.a Brain MRI at 4 months: (**E**) axial section T2-weighted, (**F**) axial section DWI (b = 1000), (**G**) coronal section T2-weighted, (**H**) sagittal T1-weighted section. The images show brainstem hypoplasia and mega cisterna magna, severe thinning of the corpus callosum, and moderate enlargement of the ventricular system and subarachnoid spaces. **I**–**L** Patient 1.a Brain MRI at 8 months: (**I**) axial section T2-weighted, (**J**) axial section DWI (b = 1000), (**H**) coronal section FLAIR T2-weighted, (**L**) sagittal section FLAIR T2-weighted. The images showing in DWI restricted signal of both thalami, hyperintense in T2 weighted images, and progressive increase in subcortical cortical atrophy. Patient 1.b Brain MRI at 11 months: (**M**) axial section T2-weighted image, (**N**) axial section DWI (b = 1000), (**O**) coronal section T2-weighted, (**P**) sagittal section T1-weighted section. The images show mild thinning of the corpus callosum, mild hypoplasia of the brainstem, and moderately wide cisterna magna, ventricular system, and subarachnoid spaces.

**Fig. 2:**
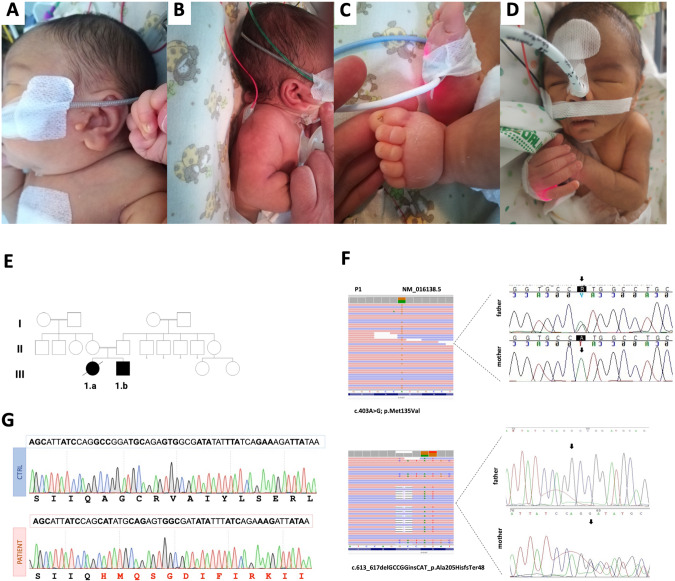
Genetic diagnosis. Pictures representative of dysmorphisms present in P1.a (**A**–**C**) and P1.b (**D**); **E** Family’s pedigree; **F** Exome analysis showing the c.613_617delGCCGGinsCAT (p.Ala205HisfsTer48) and c.403A>G (p.Met135Val) variants in P1.a and their segregation in parents’ DNA; **G** electropherogram of the allele carrying the variant c.613_617delGCCGGinsCAT showing the reading frame error in comparison with control.

A consanguineous Bangladesh family was followed in our center for a first pregnancy with a female fetus (P1.a) showing at 12 weeks of gestation increased fetal nuchal translucency (up to 6 mm) and bilateral choroid plexus cysts. Karyotype and CGH-array were performed on chorionic villus samples with no alteration in the chromosomal number or structure (46, XX). At 20 weeks of gestation, the ultrasound identified cystic hygroma, dysmorphic profile, diffuse edema in soft tissues and lower extremities, mild cardiomegaly, hyperechoic bowel with dilated loop, and amniotic fluid at lower limits. Prenatal genetic testing of a RASopathy-related genes panel resulted in negative, as well as the study of the most frequent CFTR mutations in both parents. At 32 weeks of gestation, the fetal ultrasound examination revealed intra-uterine growth retardation (IUGR, <5° centile), oligohydramnios, mild cardiomegaly with left ventricular hypertrophy, reduced caliber of the aortic arch, intestinal hyperechogenicity with moderate dilatation (Fig. 1A–C). A dysmorphic profile with lower extremity abnormalities was also described. She was born at 35^+6^ weeks of gestational age by emergency caesarian section for preterm premature rupture of membrane (PPROM). At birth, her APGAR scores were 5 and 10 at 1 and 5 min, respectively, and she presented mild metabolic acidosis (pH: 7.28, pCO2: 53 mmHg, pO2: 5 mmHg, BE: −2.4 mmol/L, lactic acid 2.7 mmol/L) and oliguria that were treated with bicarbonate supplementation and furosemide. Dysmorphisms were noted: brachial-plagiocephaly, large anterior fontanelle, asymmetrical facies with left hyposomia, epicanthus, saddle nose, low-set, and posteriorly rotated ears with multiple bilateral preauricular tags, and a sacral dimple (Fig. 2A–C). Rib cage dysmorphisms were also found in the chest X-ray study.

On the second day of life, an intestinal X-ray showed intestinal atresia with suspected meconium peritonitis. Therefore, she underwent exploratory laparotomy: voluminous meconium ileum and the dilated intestinal tract were removed, two ileostomies were packed, and a complementary appendectomy was performed. After one month, another small, obliterated tract of bowel was removed, and soon after, a recanalization surgery with terminal ileo-ileal anastomosis was performed; a few days later, due to dehiscence of surgical anastomosis, she underwent new ileostomy packing and removal of an additional segment of intestine. Two and a half months after birth, recanalization with ileal terminus-terminal anastomosis was packed together with additional stump removal. At six months of life, repetitive episodes of bilious vomiting brought her to the ER. Abdominal X-ray showed intestinal occlusion and removal of necrotic tracts was performed. The post-surgical course was complicated by an abdominal compartment syndrome associated with kidney and liver failure, requiring intensive care and continuous renal replacement therapy (CRRT). Final recanalization with jejunum-colic terminus-terminal anastomosis was performed at 8 months.

The pathological condition resulted in poor postnatal growth, generalized hypotonia, psychomotor delay, progressive hypertrophic cardiomyopathy, ascendent aortic dilatation, arterial hypertension, kidney dysfunction with increased cortical echogenicity, episodes of desquamation and skin alteration consisting of thin, papyraceous skin, grayish-itch color with thick skin at palms and soles of feet, dysplastic toenails at feet level, anterior bowing of tibias, bilateral importable lymphedema of feet.

Metabolic investigations including plasma and urinary amino acids, urinary organic acids, plasmatic acylcarnitine, plasma and liquor lactic acid, alpha-glucosidase and beta-cerebrosidase, sialic acid, urinary oligosaccharides and mucopolysaccharides, biochemical and biophysical analysis of liquor were normal.

Brain MRI performed at 4 months documented marked thinning of the corpus callosum, moderate brainstem hypoplasia, mega-cisterna magna, and moderate enlargement of the ventricular system and subarachnoid spaces (Fig. 1E–H). At 8 months, a follow-up brain MRI showed progressive cortical-subcortical atrophy and the onset of an area of restriction on diffusion studies in the thalami (Fig. 1I-L).

A muscle biopsy was performed to investigate the mitochondrial function at 4 months of age and, immediately after, supplementation with a high dose of oral CoQ_10_ (30 mg/kg/day) was started with a positive effect on psychomotor development and skin desquamation. However, the patient suddenly passed away at 14 months with multiorgan failure during an episode of metabolic acidosis triggered by prolonged fasting at home.

Being informed of the recurrence risk of the condition, in their second pregnancy the parents opted to not perform a prenatal diagnosis; the second baby boy (P1.b) reached our attention at 18^+5^ weeks of gestation. A fetal ultrasound revealed a perimembranous ventricular septal defect. At 25^+6^ weeks of gestation, IUGR (<5° centile) was described. The ultrasound scans performed in the third trimester of pregnancy showed multisystemic alterations with severe oligohydramnios, biventricular cardiomyopathy, tricuspid valve insufficiency and pulmonary valve dysplasia, and echogenic bowel (Fig. 1D). He was born at 36 weeks of gestational age by caesarian section. Immediately after birth, he presented mixed metabolic acidosis (pH 7.18, pCO2 59.5 mmHg, pO2 40,5 mmHg, HCO_3_^−^ 18.3 mmol/L, BE −7.1 mmol/L, lactic acid 58.3 mg/dl), hypotonia, hypotension, acute kidney failure and ileal stenosis-atresia that were respectively treated with bicarbonates, dobutamine, furosemide and corrective surgery procedure with ileostomy. Kidney ultrasound revealed small asymmetrical kidneys, cortical hyperechogenicity, reduced cortico-medullary differentiation, and modest bilateral pyelic dilatation. Dysmorphic features were noted as microcephaly (head circumference <2° pc), low-set ears, left preauricular tag, thick and arched eyebrows, and inverted epicanthus (Fig. 2D). Cardiac ultrasound confirmed biventricular cardiac hypertrophy with aspects of left ventricular non-compaction cardiomyopathy, systolic function within normal limits (FE 55% at last follow-up at one year of age), and mild tricuspid insufficiency. Hearing assessment revealed bilateral sensorineural deafness. Supplementation with high-dose oral CoQ_10_ (30 mg/kg/day) and riboflavin (150 mg/day) was started from birth.

Nineteen days after birth he was subjected to a second surgical procedure with double ileostomy performed proximally to the affected sections, due to the onset of necrotizing enterocolitis of an ileal loop proximal to the afferent stoma. At three months he underwent a stoma reversal of the first couple of ileostomies, and on that occasion, a severe colonic hypotrophy was discovered with a strong suspicion of an ileocecal valve atresia. At five months, he was subjected to resection of the terminal ileum, caecum, part of the ascending colon, and ileocolic terminal-terminal anastomosis. Brain MRI was performed at 11 months, showing moderate thinning of the corpus callosum, modest hypoplasia of the brain stem, and moderately wide cisterna magna, ventricular system, and subarachnoid spaces, these latter prevalently in the frontal region. No alteration over diffusion sequenced was gathered (Fig. 1M–P).

The first 10 months of life were characterized by multiple hospitalizations, surgical procedures, and infections, which all resulted in a mild developmental delay. After this first period, he demonstrated an impressive improvement in motor and cognitive development, and his renal and cardiac functions remained stable (Video – 12 months of age). The Hammersmith Infantile Neuromotor Scale at one year of age (73/78) did not show any pathological alteration in tone, posture, movement, or reaction. At the last follow-up, at 18 months of age, he was able to walk with minimal support and to say a limited number of words.

### Muscle biopsy studies

**Fig. 3:**
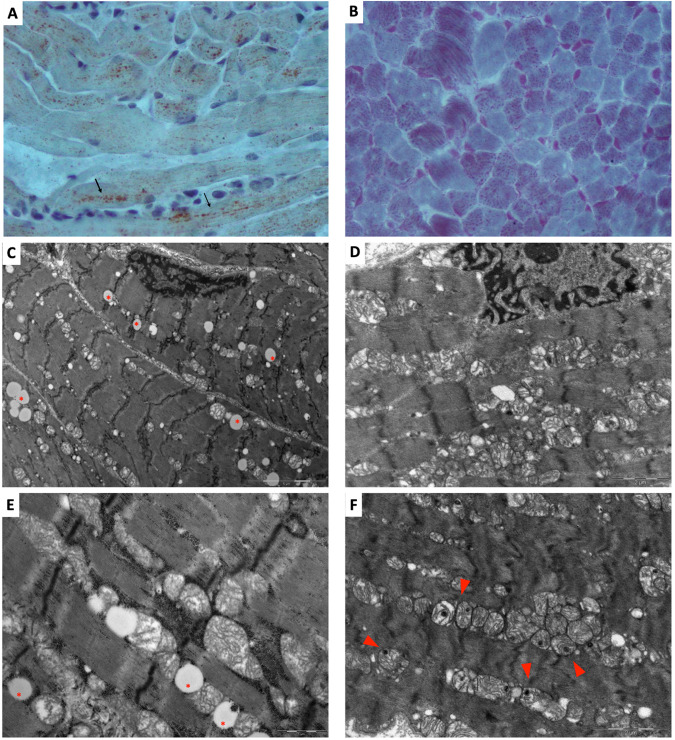
Muscle biopsy studies. **A** Oil red O stain on P1.a muscle evidencing lipid droplets organized in chain in the longitudinal fibers (black arrows); **B** Gomori’s Trichrome on P1.b muscle showing increased intermyofibrillar globular red staining; Ultrastructure images (**C** and **E**: P1.a muscle; **D** and **F**: P1.b muscle) showing lipid droplets (red stars) and damaged mitochondria, with swollen mitochondria, matrix extraction, cristae fragmentation and globular electron-dense granular inclusions (red arrowhead).

Histological stains on P1.a and P1.b muscle biopsy showed mild myogenic damage, with increased lipid (Fig. 3A) and increased intermyofibrillar stain with Gomori’s Trichrome (Fig. 3B), suggesting a mitochondrial abnormality. Histochemical stains for oxidative enzymes showed no significant alterations (data not shown). Ultrastructural analysis revealed degenerative characteristics of mitochondria with incremented lipid rate and swollen mitochondria with matrix extraction, cristae fragmentation, and electron-dense granules in the matrix in both patients. Nuclei showed irregular profiles with deep membrane invaginations (Fig. 3C–E).

**Fig. 4:**
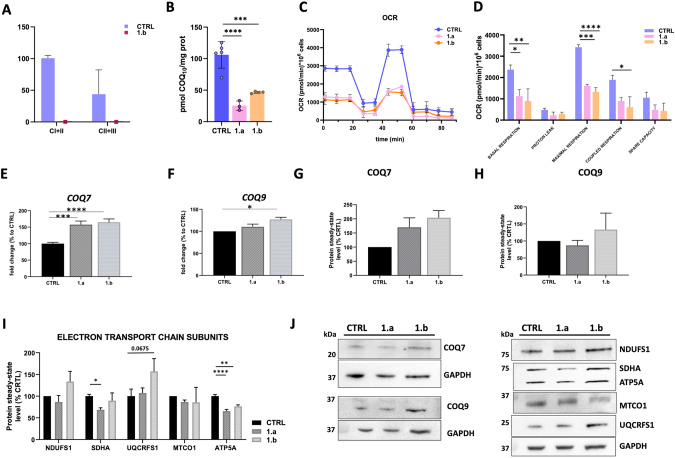
In vitro functional studies. **A** Mitochondrial Complex I + III and II + III respiratory chain activities showing abolished activities in muscle homogenate of P1.b; **B** CoQ_10_ steady-state levels analyzed by HPLC linked to the electrochemical detector, showing a dramatic reduction in fibroblasts of both patients with respect to control; **C**, **D** Seahorse analysis in patients’ and control fibroblasts confirming a general severe impairment of mitochondrial cell respiration; mRNA expression (**E**, **F**) and protein steady-state level (**G**, **H**) of *COQ7* and *COQ9* genes; **I** Steady-state level of OXPHOS proteins; **J** Representative images of western blot analysis of the steady-state levels of COQ7, COQ9 and OXPHOS subunits.

Mitochondrial biochemistry displayed an increased level of citrate synthases (CS = 234.7 nmol/min mg and 287.14 nmol/min mg, respectively in P1.a and P1.b; normal range 25–136 μmol/min/mg) while normal activities of the complexes I, II, III, and IV was found (Supplementary Table 2). Interestingly, CI + CIII and CII + CIII combined enzymatic activities performed in muscle P1.b, resulted in total absence compared to control muscles (Fig. 4A).

### Molecular genetics

Whole mitochondrial genome sequencing identified a homoplasmic variant m.8698A>G/MT-ATP6 in DNA extracted from the muscle of patient P1.a. The variant was of uncertain significance, but the segregation analysis classified it as benign since it was identified in homoplasmy in the mother’s urine.

According to our local protocol for children of consanguineous parents (Fig. 2E), the WES experiment was only performed in proband P1.a. After filtering, no pathogenic/likely pathogenic homozygous variants were identified in genes known to cause the congenital defects described in P1.a. However, two different heterozygous mutations were identified in the *COQ7* gene (NM_016138.5): an insertion/deletion (c.613_617delGCCGGinsCAT) in exon 6, predicted to result in a frameshift mutation (p.Ala205HisfsTer48), and a c.403A>G transition in exon 4, resulting in a Met135Val substitution (Fig. 2F, G).

Variants were segregated in the parents that were confirmed heterozygous carriers, for p.Ala205HisfsTer48 in the mother and p.Met135Val in the father, respectively (Fig. 2F). Sanger sequencing showed the presence of the compound heterozygous variants also in the affected brother P1.b.

Both frameshift and missense variants were found in the South Asian population according to the gnomAD v4.0 database with a low frequency of 0.0002791 and 0.0001184, respectively. However, no homozygous individuals for these variants have been identified.

The amino acid residue of p.Met135Val is located in a highly evolutionary conserved region, as demonstrated by multiple alignments of 100 vertebrate sequences in the UCSC Genome Browser with a high PhyloP score of 8.87. In silico analysis, including SIFT (score = 0), Polyphen-2 (score = 0.99), and CADD (score = 24.4) also predicted the variant to be damaging to the protein structure and function. According to ACMG classification [23], the frameshift variant was classified as “Likely pathogenic” (criteria: PVS1 and PM2); instead, the missense variant falls into the tier of “Uncertain significance” (criteria: PP3, PM2, BP1) but it can be upgraded as “Likely pathogenic” since it was detected in trans with a potentially pathogenic variant (criteria: PM3).

### In vitro studies

A statistically significant decrease in the CoQ_10_ steady-state levels was observed in patients’ fibroblasts compared to controls (Fig. 4B). Seahorse analysis was performed to assess the oxygen consumption rate (OCR) in the patient’s fibroblasts. OCR analysis unveiled that both basal and maximal respiration were significantly reduced in P1. and P1.b compared to controls. Similarly, the levels of coupled respiration were lower in patients (Fig. 4C, D).

Quantitative analysis of mRNA levels of *COQ7* and *COQ9*, a major interactor of COQ7 [24], showed a consistent increase in both patients compared to healthy controls (Fig. 4E, F). Western blot analysis of COQ7 and COQ9 proteins confirmed the trend toward an increase in patients compared to controls (Fig. 4G, H). Steady-state levels of OXPHOS subunits revealed a significant reduction in the most severe patient P1.a, while a slighter decrease was found in P1.b compared to controls for both Complexes II and V (Fig. 4I, J).

## Discussion

*COQ7* defects cause a primary CoQ_10_ deficiency associated with three main clinical phenotypes based on our recent literature review (Supplementary Table 1): encephalopathy characterized by ataxic gait, spasticity, central hypotonia, and sensory visual/hearing impairment reported in 4 patients; peripheral neuropathy presenting with axonal motor neuropathy impacting distal lower limbs with/without upper motor neuron involvement reported in 26 patients; and neonatal-onset of multisystemic disease with dysmorphisms and variable involvement of kidney, brain, gastrointestinal tract, lung and heart reported in 3 patients. There is no clear genotype-phenotype correlation, and the same variant can cause different clinical phenotypes.

Here, we report for the first time a fetal imaging study demonstrating a dysmorphic profile, cardiomegaly biventricular hypertrophic cardiomyopathy, and intestinal hyperechogenicity at the 20^th^week of gestation as first disease signs in a family with *COQ7* gene defect. Prenatal signs of oligohydramnios and IUGR were previously reported in the neonatal form of *COQ7* defect but none of the previously published patients display a major organ/tissue dysfunction. After birth, intestinal occlusions were the main debilitating symptoms in our patients, requiring multiple surgical procedures with ileostomy in the first year of life. This is the first report of gastrointestinal occlusions in CoQ_10_ deficiency. In the literature, only one patient with ADCK4-related CoQ_10_ deficiency has been reported with intestinal symptoms diagnosed as Crohn’s disease [25]. Gastrointestinal dysmotility and pseudo-occlusions are instead the main clinical symptoms of Mitochondrial NeuroGastrointestinal Encephalopathy (MNGIE) and other MNGIE-like diseases (e.g. POLG or RRM2B-related encephalopathy, MELAS) [26, 27]. The mechanism responsible for the severity of intestinal symptoms has not been clarified yet. Intestine samples were not available for biochemical studies after surgery in our patients. In vivo disease models of *COQ7* defect have demonstrated the role of the proteins in CoQ_10_ biosynthesis. *Coq7* knockout mouse model is embryonically lethal suggesting the essential role for life whereas the phenotypical characterization of clk-1 (human homologue of COQ7) mutants in the *C. Elegans* model showed a defective time in defecation suggesting a neuromotor dysfunction in the intestine [28, 29]. Further studies in patients’ biological samples and/or organoids are needed to better define the tissue-specific intestinal involvement. Additional clinical signs and symptoms in our patients were developmental delay, hypotonia, and kidney dysfunction. The first patient presented acute metabolic acidosis after a prolonged fasting state at home and died because of multiorgan failure. Diagnosis of the first proband led to early intervention with a high dose of CoQ_10_ supplementation in the second child with a positive response. At the last follow-up at 18 months of age, he was free from intestinal occlusion episodes for one year, and he recovered his motor and language milestones. Our study suggests that an accurate fetal ultrasound may identify signs of CoQ_10_ deficiency and lead to early diagnosis, allowing for rapid treatment implementation. CoQ_10_ supplementation should be administered at a high dose of 30 mg/kg/day at the earliest point in the disease history to reduce tissue damage and increase the chances of disease stabilization.

COQ7 is an iron-dependent hydroxylase that interacts with the lipid-binding auxiliary protein COQ9 in a tetramer composed of two heterodimers. COQ9 enhances the hydroxylase activity of COQ7. In fact, it has been recently demonstrated that two COQ7:COQ9 tetramers form a soluble octameric complex whose primary function is facilitating the translocation of CoQ intermediates from the bilayer to the proteins’ lipid-binding sites [24]. Pathogenic variants in both *COQ9* and *COQ7* genes cause CoQ_10_ deficiency and accumulation of the penultimate CoQ intermediate, demethoxy-CoQ (DMQ) [24].

We have identified two novel compounds heterozygous variants, an insertion/deletion c.613_617delGCCGGinsCAT in exon 6 causing a frameshift change p.Ala205HisfsTer48, and a missense variant c.403A>G transition, resulting in a Met135Val substitution.

We have demonstrated a drastic decrease of CoQ_10_ levels in the fibroblasts of both patients and the abolished combined activity of complexes I + III and complexes II + III in P1.b muscle homogenate, confirming the pathogenicity of the compound heterozygous variants in the *COQ7* gene. In vitro studies in skin-derived patients’ fibroblast cell lines also demonstrated the impact of CoQ_10_ deficiency on cell respiration, with a statistically significant reduction in both basal and maximal respiration.

A previous study demonstrated that amorphic variants cause loss of COQ7 protein and consequently neonatal onset of severe CoQ_10_ reduction, while hypomorphic variant causes a milder reduction of COQ7 protein associated with a late-onset phenotype [30]. In our study, quantitative analysis of *COQ7* gene expression and protein steady-state level demonstrated a statistically significant increase in mRNA levels and a trend toward an increase of COQ7 protein level in patients’ fibroblasts compared to controls. One of our variants affects the amino acid residue Ala205, one of the contact sites between the α6 helix of COQ7 and α7 helix of COQ9 at the N-terminal [24]. Therefore, we analyzed *COQ9* gene expression and protein level and, similarly to COQ7, we found an increase in both patients with respect to control. Based on this observation, we can hypothesize that the frameshift variant c.613_617delGCCGGinsCAT, which produces a small terminal protein truncation, would affect the stability of the COQ7-COQ9 tetramer and would be responsible for a compensatory increase in the expression of both proteins. We cannot rule out the possibility that the c.403A>G point mutation would also contribute to the induction of a compensatory increased expression of COQ7. Nonetheless, an overall compensatory mitochondrial biogenesis suggested by the increase in the citrate synthase activity could also reflect a response to the general respiratory defect observed by Seahorse respirometry.

In conclusion, our study enlarged the clinical spectrum of *COQ7* defects and identified antenatal clinical signs of the disease that can prompt early diagnosis and treatment supplementation with a positive outcome.

## Supplementary information

Video

Supplementary Table 1

Supplementary Table 2

## Data Availability

The authors confirm that the data supporting the findings of this study are available within the article and its supplementary materials.

## Code availability

Additional data are available from the corresponding author upon reasonable request.
