# Supplementary material for: COQ7 defect causes prenatal onset of mitochondrial CoQ10 deficiency with cardiomyopathy and gastrointestinal obstruction
Source: Eur J Hum Genet. 2024 May 3;32(8):938–46. doi: 10.1038/s41431-024-01615-w (PMC11291740; doi:10.1038/s41431-024-01615-w)
Supplement: Supplementary file 2 — Supplementary Table 1 [file 41431_2024_1615_MOESM2_ESM.docx]

| **Supplementary Table 1.a Peripheral neuropathy** | | | | | | | | | | | | | |
| --- | --- | --- | --- | --- | --- | --- | --- | --- | --- | --- | --- | --- | --- |
| **Variants** (NM_016138.5) | **Age at Onset** | **Type of neuropathy** | | **Limb weakness** | **Muscle atrophy** | **Walking difficulties** | **Pes cavus** | **Ataxia** | **UMN involvement *** | **Hearing loss** | **Additional**  **signs/symptoms** | **Neuroimaging** | **References** |
|  |  | **Axonal** | **Motor/Sentitive** |  |  |  |  |  |  |  |  |  |  |
| c.1A>G; p.Met1? | 5y | + | +/- | + | + | + | + | - | + | - | Seizures | Normal Brain and Spine MRI | Rebelo et al, 2023 |
| c.1A>G; p.Met1? | 10y | + | +/- | + | + | + | + | - | + | - | Distal reduced pinprick perception  Tremor | Normal Brain and Spine MRI | Rebelo et al, 2023 |
| c.1A>G; p.Met1? | 1y | + | +/- | + | + | + | + | + | + | - | ID | Mild cerebellar atrophy | Rebelo et al, 2023 |
| c.1A>G; p.Met1? | school age | + | +/- | + | + | + | + | + | + | + | Distal reduced vibration perception  Learning difficulties | Cerebellar atrophy. | Rebelo et al, 2023 |
| c.1A>G; p.Met1? | 4y | + | +/- | + | + | + | - | + | + | - | Distal reduced vibration perception | NA | Rebelo et al, 2023 |
| c.1A>G; p.Met1? | 10y | + | +/- | + | + | + | - | + | + | - | Distal reduced vibration perception | NA | Rebelo et al, 2023 |
| c.1A>G; p.Met1? | 10y | + | +/- | + | + | + | NA | - | - | + | - | NA | Smith et al, 2023 |
| c.1A>G; p.Met1? | 10y | + | +/- | + | + | + | NA | - | - | - | - | NA | Smith et al, 2023 |
| c.1A>G; p.Met1? | 10y | + | +/- | + | + | + | NA | - | - | + | - | Normal Brain MRI | Smith et al, 2023 |
| c.3G>T; p.1Met? | 12y | + | +/- | + | + | + | + | - | + | NA | - | Normal Brain MRI | Jaquier et al, 2023 |
| c.3G>T; p.1Met? | 9y | + | +/- | + | - | + | + | - | + | + | Distal reduced thermic perception | NA | Jaquier et al, 2023 |
| c.3G>T; p.1Met? | 10y | + | NA | + | + | + | + | - | - | NA | - | NA | Jaquier et al, 2023 |
| c.3G > T; p.Met1? | 5y | + | +/- | + | + | + | NA | + | + | - | Mild NDD and ID; | NA | Wongkittichote et al, 2023 |
| c.160C>T; p.Arg54Trp;  c.467T>G; p.Leu156Arg | 15y | + | +/- | + | + | + | + | + | + | NA | - | NA | Liu et al, 2023 |
| c.161G > A; p.Arg54Gln | 3y | + | +/- | + | + | + | NA | NA | + | - | Mild NDD and ID; | Lateral ventricles enlargement, PVWM thinning | Wongkittichote et al, 2023 |
| c.161G > A; p.Arg54Gln | 3y | + | +/- | + | - | + | NA | - | + | - | - | Corpus callosum thinning | Wongkittichote et al, 2023 |
| c.161G > A; p.Arg54Gln | 3y | + | +/- | + | + | + | + | - | + | - | Distal reduced vibration perception | Normal Brain and spine MRI | Rebelo et al, 2023 |
| c.197T>A; p.Ile66Asn  c.446A>G; p.Tyr149Cys | NA | + | NA | NA | NA | NA | NA | NA | NA | NA | Mild  neurodegenerative disorder | NA | Theunissen et al, 2018 |
| c.197T>A; p.Ile66Asn  c.446A>G; p.Tyr149Cys | NA | + | NA | NA | NA | NA | NA | NA | NA | NA | Mild  neurodegenerative disorder | NA | Theunissen et al, 2018 |
| c.197T>A; p.Ile66Asp;  c.446A>G; p.Tyr149Cys | mid-teens | + | +/- with prox. involvement | + | + | + | - | NA | - | - | Distal reduced pinprick perception | NA | Rebelo et al, 2023 |
| c.197T>A; p.Ile66Asp;  c.446A>G; p.Tyr149Cys | early childhood | + | +/- with prox. involvement | + | + | + | - | NA | - | - | NA | NA | Rebelo et al, 2023 |
| c.197T>A; p.Ile66Asp;  c.319C>T; p.Arg107Trp | <10y | + | +/- | + | + | + | + | - | + | + | Tremor  Distal reduced vibration perception | NA | Rebelo et al, 2023 |
| c.253-2A>T;  c.467T>A p.Leu156Glu | 8y | + | +/- | + | + | + | + | - | - | NA |  | NA | Liu et al, 2023 |
| c.319C>T; p.Arg107Trp | 8-10y | + | +/+ | + | + | + | - | - | + | + | Distal reduced pinprick perception | NA | Rebelo et al, 2023 |
| c.446A > G; p.Tyr149Cys;  c.3G > T; p.Met1? | 12y | + | +/- | + | + | - | NA | - | - | - | Learning difficulties | Normal Brain MRI | Wongkittichote et al, 2023 |
| c.467T>G; p.Leu156Arg  c.599_600delAGinsTAATGCAT; p.Lys200IlefsTer56 | 6y | + | +/+ | + | + | + | + | - | NA | - | - | NA | Zhang et al, 2023 |

| **Supplementary Table 1.b Encephalopathy** | | | | | | | | | | |
| --- | --- | --- | --- | --- | --- | --- | --- | --- | --- | --- |
| **Variants** (NM_016138.5) | **Onset (years)** | **NDD** | **Hypotonia** | **Spasticity** | **Muscle weakness** | **Walking difficulties** | **Ataxia** | **Sensorineural visual/hearing loss** | **Neuroimaging** | **References** |
| c.161G>A; p.Arg54Gln | 1 | + | + | + | + | + | + | NA | Hyperintensities in supratentorial bilateral periventricular WM | Wang et al, 2022 |
| c.332T>C; p.Leu111Pro;  c.308C>T; p.Thr103Met | 1 | + | + | + (progressive) | + | + | - | NA | Normal Brain MRI | Wang et al, 2017 |
| c.332T>C; p.Leu111Pro;  c.308C>T; p.Thr103Met | 2 | NA | NA | + (progressive) | + | + | - | -/+ | Normal Brain MRI | Hashemi et al, 2021 |
| c.332T>C: p.Leu111Pro | 1 | - | - | + (progressive) | + | + | - | -/+ | Normal Brain MRI | Sadr et al, 2023 |

| **Supplementary Table 1.c Multi-systemic disease** | | | | | | | | | | | |
| --- | --- | --- | --- | --- | --- | --- | --- | --- | --- | --- | --- |
| **Variants** (NM_016138.5) | **Prenatal-onset (signs)** | **GI** | **Lung** | **Heart** | **Kidney** | **SNC** | **Muscle** | **Sensorineural Visual/Hearing loss** | **Dysmorphisms** | **Neuroimaging** | **References** |
| c.161G > A; p.Arg54Gln;  c.446A > G; p.Tyr149Cys | Oligohydramnios | Feeding difficulties | Hypoplasia Pneumothorax | AA dilatation | - | NDD  Hypotonia  UMN involvement* | Muscle atrophy  Limb weakness | +/+ | - | Delayed myelination, increased signal in the pontine tegmentum, left posterior temporal infarction. Elevated lactate peak. | Wongkittichote et al, 2023 |
| c.422T>A; p.Val141Glu | Oligohydramnios  IUGR | Feeding difficulties | Hypoplasia;  Pulmonary hypertension | hCMP  Systemic hypertension | Dysplasia | NDD  Hypotonia  UMN involvement*  Infantile spasms | Muscle atrophy  Limb weakness  Ptosis | +/+ | + | Normal Brain MRI | Freyer et al, 2015 |
| c.599_600delinsTAATGCATC; p.(Lys200Ilefs*56;  c. .319C>T; p.(Arg107Trp | Oligohydramnios IUGR  CM | Feeding difficulties | Central hypoventilation | hCMP,  Tricuspid valve regurgitation, pericardial effusion | Dysplasia cystes | NDD  Hypotonia  UMN involvement* | Muscle atrophy  Limb weakness | +/+ | + | Atrophy, periventricular leukomalacia, lacunar infarcts, basal ganglia and thalami hypodensities with lactate peaks | Kwong et al, 2019 |
| c.613_617delGCCGGincCAT; p.Ala205HisfsTer48;  c.334A>G; p.Met112Val | Oligohydramnios IUGR  CM  intestinal hyperechogenicity with dilatation | Ileal stenosis-atresia  recurrent bowel obstruction | - | hCMP,  Systemic hypertension  AA dilatation | Dysplasia acute renal failure | NDD  Hypotonia  UMN involvement* | Limb weakness  Muscle atrophy | -/+ | + | Thin CC, brainstem hypoplasia, neurodegenerative processes with progressive cerebral atrophy and an area of restriction on diffusion studies in the bi-thalamic area extended posteriorly to the pons | Present study |
| c.613_617delGCCGGincCAT; p.Ala205HisfsTer48;  c.334A>G; p.Met112Val | Oligohydramnios IUGR  CM  intestinal hyperechogenicity with dilatation | Ileal stenosis-atresia  recurrent bowel obstruction | - | hCMP  Tricuspid valve regurgitation | Dysplasia; chronic kidney disease | NDD  Hypotonia  UMN involvement* | Muscle atrophy | -/+ | + | Thin CC, brain stem hypoplasia, and moderately wide cisterna magna, ventricular system, and subarachnoid spaces | Present study |

AA= ascending aorta; CM=cardiomegaly; ID= intellectual disability; hCMP= hypertrophic cardiomyopathy; MRI=magnetic resonance imaging; N=normal; NA=not-available; NCS=nerve conduction study; NDD= neurodevelopmental delay; WM=white matter; Y=years; UMN: upper motor neuron; *upper motor neuron involvement including both spasticity and pyramidal signs.
