# Supplementary material for: COQ7 defect causes prenatal onset of mitochondrial CoQ10 deficiency with cardiomyopathy and gastrointestinal obstruction
Source: Eur J Hum Genet. 2024 May 3;32(8):938–46. doi: 10.1038/s41431-024-01615-w (PMC11291740; doi:10.1038/s41431-024-01615-w)
Supplement: Supplementary file 3 — Supplementary Table 2 [file 41431_2024_1615_MOESM3_ESM.docx]

**Supplementary Table 2**. Mitochondrial respiratory chain activities in muscle homogenate. Data expressed in μmol/min/mg

|  | P1.a | P1.b | Normal range |
| --- | --- | --- | --- |
| Complex I | 12 | 20.6 | 11.0-66.0 |
| Complex II | 44.7 | 56.4 | 5.8-79.4 |
| Complex III | 89 | 172.2 | 55-515 |
| Complex IV | 26 | 20.7 | 20-117 |
| Citrate synthetase | 234.7 | 287.14 | 25-136 |
